# Associations of environment and lifestyle factors with suboptimal health status: a population-based cross-sectional study in urban China

**DOI:** 10.1186/s12992-021-00736-x

**Published:** 2021-07-28

**Authors:** Yunlian Xue, Zhuomin Huang, Guihao Liu, Zicheng Zhang, Yefang Feng, Mengyao Xu, Lijie Jiang, Wenyuan Li, Jun Xu

**Affiliations:** 1 Department of Operation Management, Nanfang Hospital, Southern Medical University, 1023 Shatai South Road, Baiyun District, Guangzhou, GD 20 Guangdong Province China; 2 Guangdong Provincial People’s Hospital (Guangdong Academy of Medical Sciences), Guangzhou, Guangdong Province China; 3 School of Health Services Management, Southern Medical University, Guangzhou, Guangdong Province China; 4 Department of Hospital Administrative Office, Nanfang Hospital, Southern Medical University, 1023 Shatai South Road, Baiyun District, Guangzhou, GD 20 Guangdong Province China

**Keywords:** Suboptimal health status, Lifestyle behaviors, Environment, Urban residents, China

## Abstract

**Introduction:**

Suboptimal health status (SHS), an intermediate state between chronic disease and health, is characterized by chronic fatigue, non-specific pain, headaches, dizziness, anxiety, depression, and functional system disorders with a high prevalence worldwide. Although some lifestyle factors (e.g. smoking, alcohol consumption, physical exercise) and environmental factors (e.g. air quality, noise, living conditions) have already been studied, few studies can comprehensively illustrate the associations of lifestyle and environment factors with general, physical, mental, and social SHS.

**Methods:**

A cross-sectional study was conducted among 6750 urban residents aged 14 years or over in five random cities from September 2017 to September 2018 through face-to-face questionnaires. There were 5881 valid questionnaires with a response rate of 87%. A general linear model and structural equation model were developed to quantify the effects of lifestyle behaviors and environment factors on SHS.

**Results:**

The detection rates of general, physical, mental, and social SHS were 66.7, 67.0, 65.5, and 70.0%, respectively. Good lifestyle behaviors and favorable environment factors positively affected SHS (*P* < 0.001). Lifestyle behaviors had the largest effect on physical SHS (*β* = − 0.418), but the least on social SHS (*β* = − 0.274). Environment factors had the largest effect on mental SHS (*β* = 0.286), but the least on physical SHS (*β* = 0.225).

**Conclusions:**

Lifestyle behaviors and environment factors were important influencing factors of SHS. Physical SHS was more associated with lifestyle. Lifestyle and environment were similarly associated with mental and social SHS.

## Introduction

Health was defined by the World Health Organization (WHO) in 1946 as “a state of complete physical, mental, social well-being and not merely the absence of disease or infirmity” [1, 2]. Noncommunicable diseases (NCDs), also known as chronic disease, are the opposite side of the spectrum, which is a great challenge to health. It is reported that NCDs accounted for an estimated 80% of total deaths, responsible for 70% of all disability-adjusted life-years (DALYs) in the early twentieth century [3]. The prevalence of NCDs steadily increases with urbanization and aging [4], and more than 88% of total deaths occurred from NCDs during 2019 in China [5]. A study found that NCDs accounted for 18 of the leading 20 causes of age-standardized years lived with disability worldwide [6]. The preclinical status of NCDs and their early detection have become major issues in the promotion of basic health services during health care reform [7].

Some studies have shown that suboptimal health status (SHS) may contribute to the progression or development of chronic disease [8, 9]. SHS is a state between chronic disease and health characterized by chronic fatigue, non-specific pain (e.g., back and chest pain), headaches, dizziness, anxiety, depression, and functional system disorders [8]. In recent decades, China’urbanization has developed rapidly, with the proportion of the urban population increasing from 17.9% in 1978 to 58.5% in 2017 [10]. The rapid environmental changes accompanied by urbanization have led to the increased prevalence of major risk factors for SHS, including poor dietary habits, work stress, physical inactivity, poor breakfast eating habits, smoking, tobacco use, air pollution, and noise [2, 11, 12]. These risk factors can be categorized into two aspects: lifestyle behaviors and environment factors. Although previous studies have noted the interaction between lifestyle behaviors, environment factors and SHS [8, 9, 11, 13], the associative strengths between the factors with SHS have not been well elucidated.

SHS has a prevalence of higher than 65% in China [13–16] and has become a severe issue in many other countries [17, 18]. Moreover, the prevalence may be severely underestimated since many individuals are not aware that they are suffering from SHS. In an investigation of 6000 Chinese self-reported “healthy people,” 72.8% were in SHS [19] (see Appendix Table 6). Identification of the risk factors is essential to prevention of SHS, and would provide useful information for first-level prevention of NCDs.

This study aimed to examine the associations between lifestyle behaviors and environment factors with general, physical, mental, and social SHS in a large urban population.

## Methods

### Study design and population

We conducted a multi-city cross-sectional survey using a four-stage stratified sampling method from September 2017 to September 2018. In the first stage, we selected Guangdong Province (Southeast China), Harbin City, Heilongjiang Province (Northeast China), Sichuan Province (Northwest China), Tianjin City (East China), and Lanzhou City, Gansu Province (Southwest China) as representatives of different Chinese regions based on their geographic distribution, economic characteristics, and populational demographics. The second stage included sampling of 3 ~ 5 representative cities in each province based on demographic, economic, and geographic factors of which two cities were randomly selected in the next stage, respectively. In the final stage, 1 ~ 3 streets were randomly selected from each city and the residents were selected using sampling method, who were administrated questionnaires. To ensure representativeness, the participants on each street were stratified by male and female respondents and age (i.e., brackets of 14–24, 25–34, 35–44, 45–54, and 55+). As such, survey participants were representative of the level of SHS in their respective urban areas.

Oral informed consent was obtained from each participant prior to the data collection. This consent was deemed sufficient as participants volunteered participation and were told they still could withdraw. All data were kept strictly confidential. This experiment has obtained approval of the Ethics Committee of Nanfang Hospital (Approval number: NFEC-2019-196).

### Survey instrument

This study used an SHS questionnaire to investigate urban Chinese residents. It contained two sections (i.e. both a self-designed and standardized questionnaire) [14]. The self-designed questionnaire asked for general demographic characteristics as well as information on lifestyle and environment. Here, the demographic characteristics included age, gender, and marital status and there were ten lifestyle variables (i.e., smoking, second-hand smoke, alcohol consumption, bad dietary habits, breakfast consumption, sun exposure, physical exercise, early bedtime (before 11 pm), sleep duration, and surfing the internet), and eight environmental variables (i.e., air quality, noise, housing conditions, living conditions, neighborhood harmony, fitness facilities, and supporting facilities). All variables were reported themselves in recent 3 months. The standardized components were based upon the Sub-Health Measurement Scale V1.0 (SHMS V1.0), which were designed by our research group to assess participant health status [20]. Uniform instructions were provided by trained investigators. Each participant was asked to complete the questionnaire in approximately 25 min.

### SHS assessment

Health-status assessments were performed in accordance with the SHMS V1.0. Testing procedures revealed that it had high reliability and validity (Cronbach’s α and split-half reliability coefficients of 0.917 and 0.831, respectively) [20]. The SHMS V1.0 consists of 39 items in total. Respondents were asked to answer each of these items according to a 5-point scale (1 to 5, from very bad to very good). The SHMS V1.0 was used to assess general SHS (GS) based on three symptom dimensions, including physical SHS (PS), mental SHS (MS), and social SHS (SS). Of the 39 items, Nos. 4–12, 15, 20–25, 28, and 38–39 were reverse scored (six plus the original score). The original subscale score was the sum of all items: higher scores indicated better health status. We calculated and analyzed transformed scores to further understand and compare the data. Transformed scores were determined using the formula: (original score - theoretically lowest score) / (theoretically highest score - theoretically lowest score) × 100. Following our previous study, SHS prevalence was calculated based on transformed scores [21].

### Statistical analysis

Considering that difference provinces may have different rate of sub-health, the generalized linear mixed models (GLMM) were established to analyze the group effect of sampling areas. The intra-class correlation coefficient (ICC) close to 0 and 95% confidence interval (95%CI) indicated no significant group effect and general regression model rather than multilevel model, suggesting that the GLMM model could be used in the association analysis. A general linear model was used to analyze the association of lifestyles (environmental factors) and SHS as adjusted by demographic characteristics and environment factors (lifestyles). Furthermore, a path model of latent variables was constructed based on a hypothesized relationship between items. Structural equation modeling (SEM) was then used to analyze the complexity of associations between lifestyle factors, environment factors, and SHS to estimate model fitness and analyze the direct and indirect effects of the multiple factors used in the hypothesized model [22]. Model fitness was assessed using the five indices commonly applied in SEM analyses (i.e., the relative *X*^2^ (CMIN/DF), root-mean-square error of approximation (RMSEA), comparative fit index (CFI), goodness-of-fit index (GFI), and adjusted goodness-of-fit index (AGFI)) [23]. The bootstrapping method [22] of repeat sampling (i.e., 2000 times) was applied to verify statistical significance and calculate confidence intervals for the direct, indirect, and total effects (*P* < 0.05). All statistical analyses were conducted using (SPSS Statistics version 20.0, SPSS Inc., Chicago, IL). Two-tailed *p*-values < 0.05 were considered statistically significant.

## Results

### Participant demographic characteristics

This study surveyed 6750 urban Chinese residents aged 14 year old or more who had lived in an area for the preceding over 6 months. A total of 807 participants who had become ill during 1 month of the study period were excluded. As such, 5943 respondents were either healthy or SHS for at least a period of 1 month prior to the study. However, 62 of these surveys had missing values for lifestyle, environment, and/or SHS items, and were thus excluded. Therefore, 5881 urban residents were finally included in the current study (a valid response rate of 87.13%).

Table 1 presents overall baseline, lifestyle, and environmental characteristics. The participants included 2817 males and 3064 females with a mean age of 40.27 ± 15.69 years. Most participants were married (64.50%). Furthermore, 66.7% were in GS, 67.0% were in PS, 65.5% were in MS, and 70.0% were in SS.

**Table 1:** Frequency distribution of participant demographic characteristics (*n* = 5881)

Characteristic	*N*	*GS* mean (SD)	*PS* mean (SD)	*MS* mean (SD)	*SS* mean (SD)
Gender					
Male	2817 (47.9)	71.24 (12.65)	68.13 (14.6)	61.39 (16.03)	67.64 (12.11)
Female	3064 (52.1)	70.93 (12.75)	66.05 (14.58)	61.56 (15.3)	66.85 (11.95)
Age					
14–24	1129 (19.2)	72.65 (12.19)	65.01 (13.87)	60.85 (15.82)	72.65 (12.19)
25–34	1167 (19.84)	72.21 (12.76)	65.97 (14.93)	60.92 (15.61)	72.21 (12.76)
35–44	1345 (22.87)	71.28 (12.96)	67.3 (14.61)	61.48 (15.22)	71.28 (12.96)
45–54	1120 (19.04)	70.05 (12.08)	68.56 (14.32)	62.57 (15.5)	70.05 (12.08)
≥ 55	1120 (19.04)	69.54 (12.92)	69 (15.04)	61.53 (15.69)	69.54 (12.92)
Marital status					
Unmarried	1673 (28.45)	72.25 (12.31)	64.96 (14.31)	60.51 (15.64)	66.73 (11.64)
Married	3793 (64.5)	71.03 (12.69)	68.42 (14.51)	62.16 (15.38)	67.85 (11.99)
Divorced	200 (3.4)	66.79 (13.15)	62.7 (14.4)	58.57 (18.02)	63.28 (13.11)
Widowed	183 (3.11)	66.84 (13.75)	63.24 (16.18)	60.17 (17.38)	63.89 (13.32)
Information missing	32 (0.54)	67.24 (13.96)	61.33 (14.22)	56.34 (16.91)	62.41 (12.54)

*Note: GS* general suboptimal health status, *PS* physical suboptimal health status, *MS* mental suboptimal health status, *SS* social suboptimal health status

### Comparison of SHS for different lifestyle and environment factors

The mean standard deviation (SD) transformed scores for GS, PS, MS, and SS were 67.23 (12.03), 71.08 (12.70), 67.04 (14.63), 61.47 (15.65), respectively. The GLMM model analysis found that the group effect of provinces investigated was insignificant in the analysis of overall sub-health (ICC = 0.019, 95%CI: − 0.018 - 0.057), physical sub-health (ICC = 0.028, 95%CI: − 0.024 - 0.08), psychological sub-health (ICC = 0.011, 95%CI: − 0.011 - 0.033) and social sub-health (ICC = 0.016, 95%CI: − 0.017 - 0.048). So, the general linear model could be an appropriate measure of the association between lifestyle and environment factors with SHS.

Association between each lifestyle factor and SHS was adjusted by other lifestyle behaviors, demographic characteristics, and environment factors (Table 2). Participants who never smoked, had good dietary habits, consumed breakfast daily, did daily physical exercise, and slept 7–9 h per night had the highest GS, PS, MS, and SS transformed scores. Participants who were not exposed to second-hand smoke and never consumed alcohol had the highest GS, PS, and MS transformed scores. Participants with sufficicent sleeping (bedtimes before 11 p.m.) had the highest GS and PS transformed scores. Sun exposure was only associated with PS; the highest PS scores were found in the people with 14 h or more of sun exposure each week. Surfing the internet was only associated with MS; those who surfed less than 3 hours a day had the highest scores.

**Table 2:** Comparison of SHS with different lifestyle behaviors (mean (SD))

Variables	*N*	GS		PS		MS		SS	
		mean (SD)	*P* value	mean (SD)	*P* value	mean (SD)	*P* value	mean (SD)	*P* value
Smoking			< 0.001		< 0.001		0.001		< 0.001
Never	4079	67.67 (11.80)		71.70 (12.50)		67.15 (14.55)		62.10 (15.26)	
Quit	615	64.79 (13.06)		68.37 (13.57)		65.37 (15.11)		58.44 (17.40)	
Yes	1187	66.95 (12.12)		70.35 (12.70)		67.53 (14.60)		60.90 (15.86)	
Second-hand smoking influence			< 0.001		< 0.001		0.001		0.376
None	1150	68.95 (12.73)		72.94 (12.99)		68.97 (15.61)		62.72 (16.88)	
Little	1804	67.54 (12.03)		71.68 (12.72)		67.31 (14.29)		61.41 (15.68)	
Some	1702	66.60 (11.21)		70.31 (12.32)		66.55 (13.62)		60.92 (14.51)	
Much	1225	66.00 (12.25)		69.52 (12.65)		65.53 (15.3)		61.17 (15.90)	
Alcohol consumption			< 0.001		< 0.001		< 0.001		0.177
Never	2166	68.41 (12.26)		72.24 (13.14)		68.29 (14.81)		62.59 (15.92)	
Occasional	3107	66.86 (11.56)		70.87 (12.02)		66.51 (14.22)		61.09 (15.13)	
A little /day	427	65.79 (13.10)		69.01 (13.72)		66.32 (15.80)		60.08 (16.64)	
Some /day	106	65.13 (11.48)		67.94 (13.05)		65.27 (13.62)		60.59 (14.84)	
Much /day	75	59.47 (14.03)		62.57 (15.03)		59.64 (16.30)		54.41 (21.06)	
Bad dietary habits			< 0.001		< 0.001		< 0.001		< 0.001
No	3396	69.93 (11.63)		73.33 (12.54)		70.23 (14.21)		64.23 (14.86)	
Yes	2485	63.53 (11.59)		68.00 (12.27)		62.68 (14.06)		57.72 (15.93)	
Breakfast consumption (days/week)			< 0.001		< 0.001		< 0.001		< 0.001
Everyday	2720	69.91 (11.75)		73.06 (12.76)		70.19 (14.53)		64.63 (14.78)	
5–6	1562	67.08 (11.39)		71.15 (11.94)		66.41 (14.11)		61.66 (14.72)	
3–4	848	62.97 (11.13)		68.08 (12.05)		61.93 (13.08)		56.43 (15.45)	
1–2	609	62.69 (12.07)		67.04 (12.62)		62.75 (14.21)		55.87 (16.81)	
Never	142	62.16 (14.29)		67.58 (15.47)		62.54 (17.10)		53.23 (20.14)	
Sunshine (hours/week)			0.071		0.008		0.170		0.504
≥ 14	476	68.96 (12.57)		72.22 (13.32)		68.71 (15.10)		64.23 (15.97)	
< 14	874	69.41 (12.12)		73.20 (13.04)		69.58 (14.20)		63.30 (15.74)	
< 7	1695	67.26 (12.14)		71.11 (12.88)		67.18 (14.69)		61.37 (15.31)	
< 3	1578	66.84 (11.84)		70.80 (12.09)		66.64 (14.47)		60.93 (15.96)	
< 1	1258	65.49 (11.54)		69.50 (12.50)		64.96 (14.53)		59.98 (15.32)	
Physical exercise (days/week)			< 0.001		< 0.001		< 0.001		< 0.001
Everyday	630	72.14 (11.89)		74.46 (12.84)		73.24 (14.82)		67.07 (15.12)	
5–6	613	68.96 (12.27)		71.93 (13.02)		68.74 (14.89)		64.63 (15.28)	
3–4	1412	67.67 (12.71)		71.57 (13.24)		67.64 (14.63)		61.62 (16.82)	
1–2	2375	66.24 (11.17)		70.57 (11.82)		65.60 (14.03)		60.36 (14.57)	
Never	851	64.36 (11.82)		68.58 (13.21)		64.25 (14.43)		57.92 (15.78)	
Bedtime before 11 pm (days/week)			0.019		0.003		0.146		0.388
Everyday	1119	70.05 (12.09)		73.00 (13.41)		70.84 (14.86)		64.43 (15.44)	
5–6	1007	68.35 (12.10)		71.61 (12.73)		68.44 (14.51)		63.17 (14.79)	
3–4	1236	66.23 (12.38)		70.11 (12.87)		65.98 (14.54)		60.52 (16.01)	
1–2	1549	66.1 (11.30)		70.48 (11.97)		65.56 (13.79)		59.98 (15.51)	
Never	970	65.87 (11.96)		70.52 (12.49)		64.92 (14.95)		59.90 (15.92)	
Sleep duration (hours)			< 0.001		< 0.001		< 0.001		< 0.001
≥ 9	503	67.91 (12.95)		71.48 (13.65)		68.26 (15.34)		61.88 (16.59)	
< 9	3792	68.51 (11.63)		72.57 (12.30)		68.18 (14.41)		62.64 (14.86)	
< 7	1402	64.85 (11.67)		68.10 (12.40)		64.82 (14.23)		59.85 (15.96)	
< 5	145	56.75 (13.13)		62.18 (12.60)		56.71 (14.61)		48.37 (18.90)	
< 3	39	57.77 (11.65)		61.77 (13.69)		59.19 (14.40)		49.64 (21.62)	
Surfing the internet (hours)			0.062		0.154		< 0.001		0.983
< 3	3019	67.78 (12.60)		71.09 (13.24)		68.39 (15.00)		61.83 (16.37)	
< 5	1739	67.06 (11.40)		71.17 (12.07)		66.51 (14.00)		61.42 (14.75)	
< 7	720	66.49 (10.86)		71.46 (11.59)		64.66 (13.94)		61.18 (14.46)	
≥ 7	403	65.07 (12.05)		69.91 (13.10)		63.53 (14.39)		59.59 (15.91)	

*Note*: *GS* general suboptimal health status, *PS* physical suboptimal health status, *MS* mental suboptimal health status, *SS* social suboptimal health status

** *P* value is statistically significant, *P*<0.01

Table 3 shows the effects of environmental factors after adjusting other environment factors, demographic characteristics, and lifestyle behaviors. Pleasant housing, harmonious neighborhoods, and convenient living conditions were positively associated with GS, PS, MS, and SS. Urban green space was positively associated with GS and SS. We also observed positive associations between fresh air and GS, PS, and MS. The presence of many fitness facilities was positively associated with GS and MS. There were no significant associations between noise and SHS. However, people with spacious homes had lower GS and MS scores.

**Table 3:** Comparison of SHS with different living environmental factors (mean (SD))

Variables		*N* (%)	GS		PS		MS		SS	
			mean (SD)	*P* value	mean (SD)	*P* value	mean (SD)	*P* value	mean (SD)	*P* value
Greenery				0.002		0.358		0.011		< 0.001
	bad	3105	65.65 (11.58)		69.99 (12.44)		65.25 (14.17)		59.42 (15.10)	
	good	2776	68.99 (12.28)		72.30 (12.88)		69.04 (14.87)		63.77 (15.94)	
Air				0.002		0.005		0.001		0.274
	bad	2977	65.72 (11.47)		69.86 (12.24)		65.21 (14.35)		59.99 (15.32)	
	fresh	2904	68.76 (12.39)		72.34 (13.04)		68.92 (14.67)		63.00 (15.85)	
Noiseless				0.788		0.159		0.477		0.905
	no	4019	66.53 (12.08)		70.40 (12.69)		66.35 (14.83)		60.75 (15.82)	
	yes	1862	68.72 (11.79)		72.54 (12.61)		68.53 (14.07)		63.03 (15.19)	
Pleasant housing				< 0.001		0.036		< 0.001		0.003
	no	824	63.54 (11.55)		68.55 (12.47)		62.34 (14.12)		57.36 (15.46)	
	yes	5057	67.83 (12.00)		71.49 (12.69)		67.81 (14.57)		62.15 (15.58)	
Spacious rooms				0.003		0.434		< 0.001		0.186
	yes	663	63.60 (10.82)		68.83 (11.92)		61.61 (13.48)		58.13 (15.11)	
	no	5218	67.69 (12.10)		71.37 (12.77)		67.73 (14.62)		61.90 (15.67)	
Neighbor harmony				< 0.001		< 0.001		< 0.001		< 0.001
	no	3351	65.47 (11.67)		69.76 (12.57)		65.02 (14.19)		59.40 (15.50)	
	yes	2530	69.55 (12.11)		72.84 (12.67)		69.72 (14.77)		64.22 (15.44)	
Fitness facility				0.001		0.058		0.001		0.001
	few	4766	66.40 (11.66)		70.44 (12.41)		66.15 (14.31)		60.46 (15.42)	
	many	1115	70.74 (12.92)		73.81 (13.53)		70.86 (15.34)		65.80 (15.90)	
Living convenience				< 0.001		< 0.001		< 0.001		< 0.001
	no	3832	65.59 (11.91)		69.68 (12.62)		65.41 (14.53)		59.45 (15.68)	
	yes	2049	70.29 (11.66)		73.70 (12.44)		70.09 (14.32)		65.25 (14.90)	

*Note: GS* general suboptimal health status, *PS* physical suboptimal health status, *MS* mental suboptimal health status, *SS* social suboptimal health status

### SEM analysis of lifestyle, environment, and SHS

**Fig. 1:**
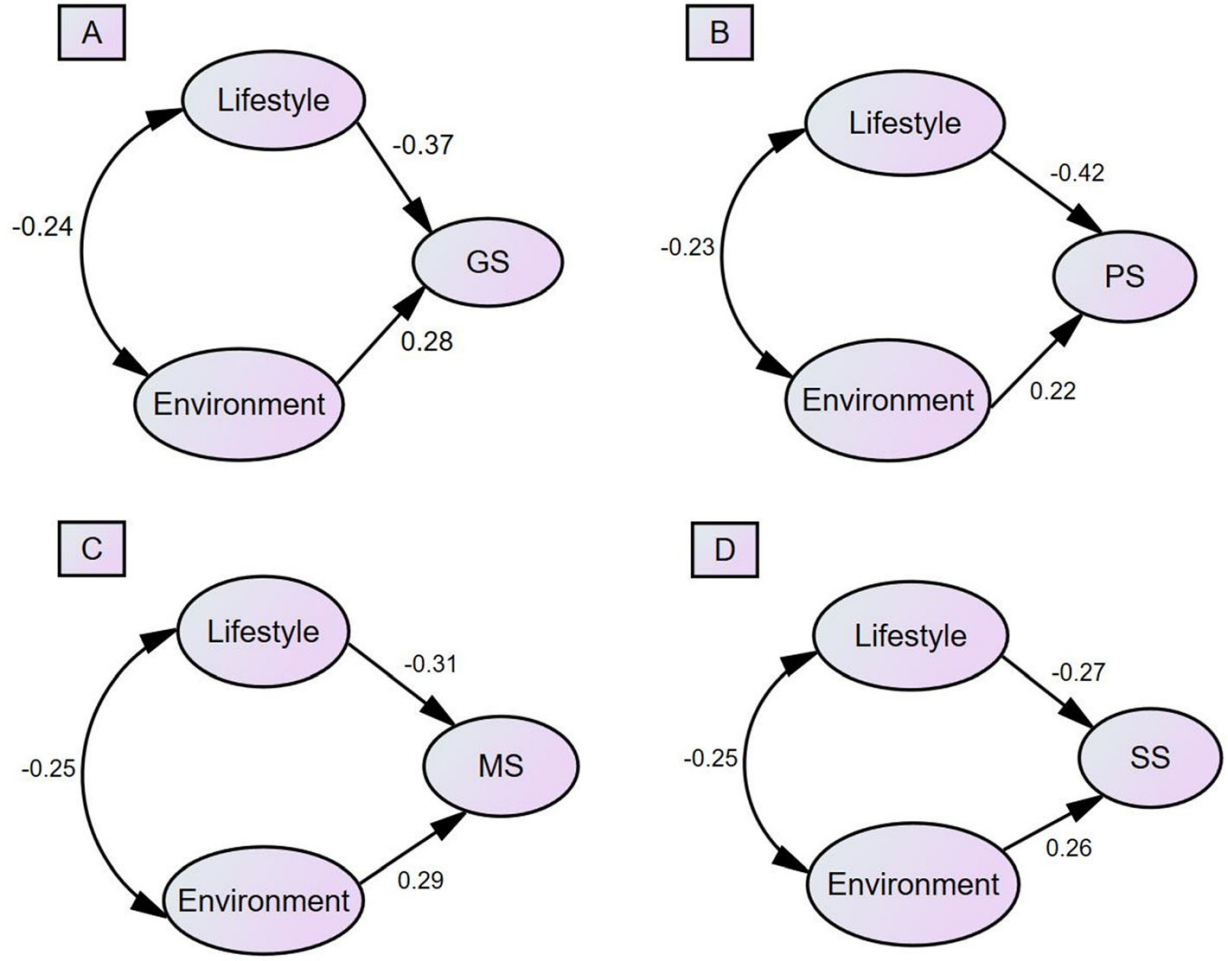
Structural equation model involving lifestyle, environment, and SHS (A = GS, B = PS, C = MS, and D = SS)

The total associations of lifestyle behaviors and environment with SHS were analyzed through SEM (Fig. 1). Although noiseless areas were not independently associated with SHS, the model was not deemed fit without an environmental noise component. The SEM thus included a “noiseless” variable. Except for the *CMIN/DF*, *CFI,* and *AGFI* of Model 1 and the *CMIN/DF* of Model 2, Table 4 presents information about the fitness measurements for all four models. The associations of lifestyle and environment factors with SHS are listed in Table 5. The data demonstrated that unhealthy lifestyle had significantly negative effects on GS, PS, MS, and SS, while good environment factors had a positive impact (*P* < 0.001). Lifestyle had the largest effect on PS (*β* = − 0.418) and the least effect on SS (*β* = − 0.274). On the other hand, environment factors had the largest effect on MS (*β* = 0.286) and the least effect on PS (*β* = 0.225). As the influencing effects were standardized, GS had a larger association with lifestyle (*β* = − 0.371), but less with environment (*β* = − 0.282); physical health was more associated with lifestyle (*β* = − 0.418), but less associated with environment (*β* = − 0.225). The associations of lifestyle behaviors and environment were similar for MS and identical for SS.

**Table 4:** Fitting effect of the structural equation models

Models	*CMIN/DF*	*CFI*	*GFI*	*AGFI*	*RMSEA*
Criterion for good fit [13]	< 5	> 0.95	> 0.95	> 0.95	< 0.05
Model 1	5.697	0.947	0.957	0.946	0.028
Model 2	5.297	0.956	0.979	0.970	0.027
Model 3	4.507	0.972	0.983	0.976	0.024
Model 4	4.269	0.974	0.986	0.980	0.024

Model 1: Structural model for lifestyle, environment, and general suboptimal health status, Model 2: Structural model for lifestyle, environment, and physical suboptimal health status, Model 3: Structural model for lifestyle, environment, and mental suboptimal health status, Model 4: Structural model for lifestyle, environment, and social suboptimal health status

**Table 5:** The associations between lifestyle, environment, and SHS as analyzed through structural equation modeling

SHS	Factors	Standardized effect	95% *CI* lower limit	95% *CI* upper limit	*P*
GS	Lifestyle	−0.371	−0.406	−0.335	< 0.001
	Environment	0.282	0.246	0.317	< 0.001
PS	Lifestyle	−0.418	−0.456	− 0.377	< 0.001
	Environment	0.225	0.182	0.265	< 0.001
MS	Lifestyle	−0.306	− 0.338	− 0.273	< 0.001
	Environment	0.286	0.252	0.321	< 0.001
SS	Lifestyle	−0.274	− 0.309	− 0.242	< 0.001
	Environment	0.263	0.227	0.299	< 0.001

## Discussion

This cross-sectional study of nationally representative urban Chinese residents found that lifestyle behaviors and environment factors were significantly associated with SHS. The associations of lifestyle behaviors with GS and PS were larger than those of environment factors. The associations of lifestyle behaviors and environment factors with MS and SS were almost identical.

To the best of our knowledge, our work was the first study on the associations of lifestyle behaviors and environment factors with SHS. The findings in this study were generally in line with those of previous studies on the relationship between lifestyle and PS [24, 25]. Innovative findings in this study were the significant associations of environment factors with MS and SS. However, the association between environment factors and mental health has been elucidated before.

This study used the 39-item SHMS V.1.0 questionnaire to analyze SHS, which includes physical, mental, and social dimensions. Our previous research indicated that SHMS V.1.0 had good internal consistency among southern Chinese medical staff members [21] and urban residents of three districts in China [26]. The detection rate of GS in Chinese urban residents was 66.7%, slightly higher than in southern China (65.1%) [16] and Tianjin (66.37%) [17].

This study found that bad lifestyle habits such as smoking, alcohol consumption, second-hand smoke exposure, poor dietary habits, and surfing the internet correlated to low SHS scores. On the other hand, healthy lifestyle habits such as breakfast eating habits, adequate sun exposure and exercise, and sufficient sleeping with a consistent bedtime before 11 pm were associated with non-SHS. A study among Chinese university students similarly depicted the correlation between a sleep duration of less than 6 hours per day and poor self-reported health problem [27]. Breakfast skipping is reported to raise risk of mortality from cardiovascular disease [28]. Furthermore, an English study reported that poor health outcomes were more common among ex-smokers and current smokers than those who had never smoked [29]. Black men with alcohol consumption and short sleep duration are more prone to poor health in the United States [30].

Accumulating evidence has indicated that exercise, physical activity, and physical-activity interventions are beneficial for physical and mental-health outcomes. Sufficient fresh air and sun exposure are also good for promoting public health [31]. In conclusion, these studies and our findings emphasize the importance of maintaining good lifestyle habits, which is a simple way to prevent SHS and improve overall well-being.

This study further found that environment factors such as sufficient greenery, fresh air, pleasant housing, less spacious rooms, harmonious neighborhoods, the presence of many fitness facilities, and convenient living conditions were associated with high SHS scores. It is well-known that positive environments (especially natural outdoor areas) are good for human health [32]. Contrary to our general expectations, however, we found that people who lived in spacious rooms were more vulnerable to both MS and GS. This may be due to feelings of emptiness in one’s surroundings. As indicated by a systematic review, living alone may be associated with low levels of positive mental health [33].

### Study strengths

This population-based study examined a sample of urban residents (5881 respondents), thus facilitating overall generalizability to the entire urban population in suboptimal health prevention in China. Furthermore, we illustrated the relative strengths of lifestyle behaviors and environment factors on the associations with SHS. We firstly illustrated the important association between environment factors and MS and SS, which had almost the same association with lifestyle behaviors. Furthermore, associated factors were examined comprehensively, including ten lifestyle behaviors and eight environment factors, which can be intervention targets and would be helpful for preventing SHS and NCDs.

### Limitations

First, because of the cross-sectional design it was not possible to confirm causal relationships of SHS with lifestyle behaviors and environment factors. Second, lifestyle factors and environmental variables were self-reported in this study, which may have potential bias and affect the accuracy of the measurement. Third, environment factors included in this study were all life-related, and other environment factors hadn’t been included. What’s more, although we have considered as many factors as possible, bias would inevitably occur because of certain factors not being included.

## Conclusions

This large-scale cross-sectional study of Chinese urban residents or more demonstrates that good lifestyle behaviors and positive environment are both associated with low rates of SHS (i.e., high SHS scores). Lifestyle behaviors are more associated with PS and GS. However, the associations of environment factors and MS and SS are greater than that with PS, which are similar with lifestyle behaviors.

## Data Availability

Data used in this study were obtained under the support of Nanfang Hospital, Southern Medical University. The ownership of the data belongs to Nanfang Hospital, Southern Medical University. Researchers who meet the criteria for access to confidential data can contact Jun Xu (drugstat@163.com) at Nanfang Hospital, Southern Medical University to request the data.

## Appendix

**Table:** **Table 6** This scale consists of 39 questions, including physical, mental and social health conditions

Sub-health measurement scale (SHMS V1.0)					
1. How is your appetite?	□ very bad	□ worse	□ average	□ better	□ very good
2. How is your sleep?	□ very bad	□ worse	□ average	□ better	□ very good
3. Are you satisfied with your hair growth?(e.g. premature graying, yellowing or hair loss)	□ very dissatisfied	□ less satisfied	□ average	□ more satisfied	□ very satisfied
4. Do you feel bitter or dry mouth?	□ never	□ few	□ sometimes	□ often	□ always
5. Do you have gastrointestinal discomfort? (e.g. acid reflux, belching, nausea (abdominal pain, bloating, diarrhea, constipation, etc.)	□ none	□ rarely	□ sometimes	□ often	□ always
6. Do you have abnormal urine symptoms? (e.g. yellow urine, painful urination, oliguria, frequent urination, excessive nocturia, etc.)	□ none	□ rarely	□ sometimes	□ often	□ always
7. Do you have head discomfort? (e.g. dizziness, headache, heavy head, head swelling, head numbness, etc.)	□ none	□ rarely	□ sometimes	□often	□ always
8. Do you have eye discomfort? (e.g. soreness, dryness, tearfulness, blurred, easily fatigued, polycythemia, etc.)	□ none	□ rarely	□ sometimes	□often	□ always
9. Do you feel discomfort of your auditory system?(e.g. tinnitus, hearing loss, ear pain, etc.)	□ none	□ rarely	□ sometimes	□often	□ always
10. Do you have difficulty bending and flexing your knees?	□ easy	□ relatively easy	□ a little difficult	□ rather difficult	□ very difficult
11. Do you have difficulty climbing 3 to 5 floors normally?	□ easy	□ relatively easy	□ a little difficult	□ rather difficult	□ very difficult
12. Do you have difficulty walking 1500 m?	□ easy	□ relatively easy	□ a little difficult	□ rather difficult	□ very difficult
13. Can your fatigue be relieved after rest?	□ never	□ rarely	□ sometimes	□ mostly can	□ absolutely can
14. Do you have enough energy to cope with daily life, work and study?	□ none	□ rarely have	□ sometimes have	□ mostly have	□ totally have
15. What state do you think your physical (somatic) health is in?	□ health	□ mild suboptimal health	□ average suboptimal health	□ severe suboptimal health	□ disease
16. Do you have confidence in yourself?	□ none	□ less confident	□ a little confident	□more confident	□ very confident
17. Are you satisfied with your current living condition?	□ very dissatisfied	□ less dissatisfied	□ average	□ more satisfied	□ very satisfied
18. Are you optimistic about the future?	□ very pessimistic	□ less pessimistic	□ average	□ more optimistic	□ very optimistic
19. Do you feel happy?	□ never	□ rarely	□ sometimes	□often	□ always
20. Do you feel nervous mentally?	□ never	□ rarely	□ sometimes	□ often	□ always
21. Do you feel bad or depressed?	□ never	□ rarely	□ sometimes	□often	□ always
22. Do you feel insecure?	□ never	□ rarely	□ sometimes	□ often	□ always
23. Do you feel scared for no reason?	□ never	□ rarely	□ sometimes	□ often	□ always
24. Do you feel lonely?	□ never	□ rarely	□ sometimes	□ often	□ always
25. Are you sensitive and suspicious?	□ never	□ rarely	□ sometimes	□ often	□ always
26. How is your memory?	□ very poor	□ worse	□ average	□ better	□ very good
27. How is your ability to think about or deal with problems?	□ very poor	□ worse	□ average	□ better	□ very good
28. What state do you think your mental health (such as emotion, cognitive ability, etc.) is in?	□ health	□ mild suboptimal health	□ average suboptimal health	□ severe suboptimal health	□ disease
29. Can you properly deal with the unpleasant things happen in your life, work and study?	□ never	□ rarely can	□ sometimes can	□ mostly can	□ absolutely can
30. Are you satisfied with your interpersonal relationship in the society?	□ very dissatisfied	□ less dissatisfied	□ average	□ more satisfied	□ very satisfied
31. Are you satisfied with your performance in life, work and study?	□ very dissatisfied	□ less dissatisfied	□ average	□ more satisfied	□ very satisfied
32. Can you adapt quickly to your new life, work and study?	□ never	□ rarely can	□ sometimes can	□ mostly can	□ absolutely can
33. Do you keep in touch with your friends and relatives frequently (such as visiting each other, telephone greetings, correspondence, etc.)?	□ never	□ rarely	□ sometimes	□ often	□ more often
34. Do you have friends you can share your happiness and sorrow with?	□ never	□ less	□ average	□ many	□ very many, more than five
35. Are there any colleagues, classmates, neighbors, relatives or friends with whom you are close to?	□ never	□ less	□ average	□ many	□ very many, more than five
36. When you need help, will your family, colleagues or friends give you material or emotional support or help?	□ never	□ rarely	□ sometimes	□ often	□ always
37. When you encounter difficulties, will you proactively seek support and help of others?	□ never	□ rarely	□ sometimes	□ often	□ always
38. What state do you think your “social health” is in (e.g. interpersonal relationship, social interaction, etc.)?	□ health	□ mild suboptimal health	□ average suboptimal health	□ severe suboptimal health	□ disease
39. What do you think your general health (including physical, mental and social health) is in?	□ health	□ mild suboptimal health	□ average suboptimal health	□ severe suboptimal health	□ disease

## Abbreviations

SHS: Suboptimal health status
GS: General suboptimal health status
PS: Physical suboptimal health status
MS: Mental suboptimal health status
SS: Social suboptimal health status
SEM: Structural equation model
CMIN/DF: relative *X*^2^
RMSEA: root mean-square error of approximation
CFI: Comparative fit index
GFI: Goodness-of-fit index
AGFI: Adjusted goodness-of-fit index
NCDs: Noncommunicable chronic diseases

## References

[1] Grad FP (2002). The preamble of the constitution of the World Health Organization. Bull World Health Organ.

[2] Bi JL, Ying H, Ya X, Cheng JR, Fei L, Tian W (2014). Association of lifestyle factors and suboptimal health status: a cross-sectional study of Chinese students. BMJ Open.

[3] Strong K, Mathers C, Leeder S, Beaglehole R (2005). Preventing chronic diseases: how many lives can we save?. Lancet..

[4] Wang L, Kong LZ, Wu F, Bai YM, Burton R (2005). Preventing chronic diseases in China. Lancet..

[5] Transcript of press conference of the office of Health China action Promotion Committee on July 31, 2019. http://www.nhc.gov.cn/xcs/s7847/201907/0d95adec49f84810a6d45a0a1e997d67.shtml.

[6] GBD 2015 Mortality and Causes of Death Collaborators (2016). Global, regional, and national life expectancy, all-cause mortality, and cause-specific mortality for 249 causes of death, 1980–2015: a systematic analysis for the Global Burden of Disease Study 2015. Lancet.

[7] Wang Y, Ge S, Yan Y, Wang A, Zhao Z, Yu X, Qiu J, Alzain MA, Wang H, Fang H (2016). China suboptimal health cohort study: Rationale, design and baseline characteristics. J Transl Med.

[8] Yan YX, Dong J, Liu YQ, Zhang J, Song MS, He Y, Wang W (2015). Association of suboptimal health status with psychosocial stress, plasma cortisol and mRNA expression of glucocorticoid receptor α/β in lymphocyte. Stress..

[9] Yan Y, Dong J, Liu Y, Yang X, Li M, Shia G (2012). Association of suboptimal health status and cardiovascular risk factors in urban Chinese workers [J]. J Urban Health.

[10] Jia RX (2018). China's 40 years of urbanization: from high speed to high quality. China Dev Watch.

[11] Chen JY, Cheng JR, Liu YY, Tang Y, Sun XM, Wang T, et al. Associations between breakfast eating habits and health-promoting lifestyle, suboptimal health status in Southern China: a population based, cross sectional study. J Transl Med. 12(1):348. 10.1186/s12967-014-0348-1 PMC4269950 25496597

[12] Ma N, Liu M (2012). Research progress on the epidemiology of sub-health state. China Prev Med.

[13] Xue YL, Xu J, Liu GH, Feng Y, Xu M, Xie J (2019). Study of association between personality and sub-health status among urban residents aged more than 14 years old in 4 cities in China. J Southern Med Univ.

[14] Xue YL, Xu J, Liu GH, Feng Y, Xu M, Xie J (2019). The mediating effect of adversity quotient on the correlation between stressful life events and sub-health status. Mod Prevent Med.

[15] Sun X, Wei M, Zhu C, Wang XL, Zhao XS, Luo R (2008). The epidemiological investigation of sub-health status in Guangdong. Shandong Med J.

[16] Xie J, Luo HB, Zhu H, Chang WJ, Xu J (2016). Prevalence and influence factors of sub-health status among urban residents in Tianjin. Chin J Public Health.

[17] Sou J, Goldenberg SM, Duff P, Nguyen P, Shoveller J, Shannon K (2017). Recent im/migration to Canada linked to unmet health needs among sex workers in Vancouver, Canada: findings of a longitudinal study. Issues Health Care Women.

[18] Dunstan RH, Sparkes DL, Roberts TK, Crompton MJ, Gottfries J, Dascombe BJ (2013). Development of a complex amino acid supplement, fatigue reviva, for oral ingestion: initial evaluations of product concept and impact on symptoms of suboptimal health in a group of males. Nutr J.

[19] Liu Z, Li M (2001). The third state and psychosomatic medicine research. Med Philos.

[20] Xu J, Feng LY, Luo R, Qiu JC, Zhang JH, Zhao XS (2011). Assessment of the reliability and validity of the sub-health status measurement scale Version1.0. J Southern Med Univ.

[21] Xu J, Xue YL, Liu GH, Feng Y, Xu M, Xie J (2019). Establishment of the norms of sub-health status measurement scale version 1.0 for Chinese urban residents. J South Med Univ.

[22] Bucy EP, Holbert RL (2014). Sourcebook for political communication research: methods, measures, and analytical techniques.

[23] Yamaga E, Sato Y, Minakuchi S (2018). A structural equation model to test a conceptual framework of oral health in Japanese edentulous patients with an item weighting method using factor score weights: a cross-sectional study. BMC Oral Health.

[24] Minich DM, Bland JS. Personalized lifestyle medicine: relevance for nutrition and lifestyle recommendations. Sci World J. 2013;129841. 10.1155/2013/129841 PMC3710624 23878520

[25] Marques A, Peralta M, Santos T, Martins J, Gaspar de Matos M (2019). Self-rated health and health-related quality of life are related with adolescents’ healthy lifestyle. Public Health.

[26] Lu Y, Xu J, Cai YJ, Xie J, Qiu JC, Wei Q (2013). Reliability and validity of sub-health status measurement scale version 1.0 for measuring the sub-health status of urban residents in three districts. China J Health Psychol.

[27] Li L, Lok KI, Mei SL, Cui XL, Li L, Ng CH, et al. Sleep duration and self-rated health in Chinese university students. Sleep Breath. 2019. 10.1007/s11325-019-01856-w 31152382

[28] Rong S, Snetselaar LG, Xu GF, Sun Y, Liu B, Wallace RB (2019). Association of skipping breakfast with cardiovascular and all-cause mortality. JAMA..

[29] Chattopadhyay K, Akagwire U, Biswas M, Moore R, Rajania G, Lewis S (2019). Role of lifestyle behaviours in the ethnic pattern of poor health outcomes in Leicester, England: analysis of a survey data set. Public Health.

[30] Jackson CL, Gaston SA, Liu R, Mukamal K, Rimm EB (2018). The relationship between alcohol drinking patterns and sleep duration among black and white men and women in the United States. Int J Environ Res Public Health.

[31] Keeling AW (2015). Historical perspectives on fresh air, sunshine, and public health. Windows Time.

[32] Zijlema WL, Christian H, Triguero-Mas M, Cirach M, van den Berg M, Maas J, Gidlow CJ, Kruize H, Wendel-Vos W, Andrušaitytė S, Grazuleviciene R, Litt J, Nieuwenhuijsen MJ (2019). Dog ownership, the natural outdoor environment and health: a cross-sectional study. BMJ Open.

[33] Tamminen N, Kettunen T, Martelin T, Reinikainen J, Solin P (2019). Living alone and positive mental health: a systematic review. Syst Rev.

